# Pulmonary Extracellular Vesicles as Mediators of Local and Systemic Inflammation

**DOI:** 10.3389/fcell.2017.00039

**Published:** 2017-04-26

**Authors:** Casper J. E. Wahlund, Anders Eklund, Johan Grunewald, Susanne Gabrielsson

**Affiliations:** ^1^Unit of Immunology and Allergy, Department of Medicine, Karolinska InstituteStockholm, Sweden; ^2^Respiratory Unit, Department of Medicine, Karolinska Institute and Karolinska University HospitalStockholm, Sweden

**Keywords:** exosomes, microvesicles, inflammation mediators, Extracellular vesicles (EVs), sarcoidosis, pulmonary, COPD, asthma

## Abstract

Cells of the airways are constantly exposed to environmental hazards including cigarette smoke, irritants, pathogens, and mechanical insults. Maintaining barrier integrity is vital, and mounting responses to threats depends on intercellular communication. Extracellular vesicles (EVs), including exosomes and microvesicles, are major signal mediators between cells, shuttling cargo in health and disease. Depending on the state of the originating cells, EVs are capable of inducing proinflammatory effects including antigen presentation, cellular migration, apoptosis induction, and inflammatory cytokine release. Cells of the airways release EVs, which can be found in bronchoalveolar lavage fluid. EVs of the airways can support inflammation in the lung, but may also exit into the circulation and carry a cocktail of pro-inflammatory molecules to recipient cells in distant organs. In this review, we discuss the possibility that EVs originating from the airways contribute to dissemination of inflammation in both lung disorders and systemic inflammatory conditions.

## Introduction

Intercellular communication is key in inducing and resolving inflammation. Extracellular vesicles (EV) are intercellular messengers present in all body fluids, transporting proteins, lipids, and nucleic acids(Raposo and Stoorvogel, 2013; Lo Cicero et al., 2015). They are involved in physiological processes at steady state and in pathological conditions, from sperm motility and coagulation to modulating diabetes, cancer and antigen-specific immunogenicity, or tolerance(Raposo and Stoorvogel, 2013; Robbins and Morelli, 2014). EVs are also implicated in inflammatory lung disorders including sarcoidosis, asthma and chronic obstructive pulmonary disorder (COPD), and may be a universal disseminator of inflammation. The two most studied EV species are endosome-originating exosomes (Kowal et al., 2014), and cell surface-shed microvesicles (MV; Cocucci and Meldolesi, 2015), and in this review we discuss how pulmonary EVs support inflammation in the lungs, but also how they may exit the lungs and contribute to dissemination of inflammation.

Gas exchange depends on large air-to-blood contact surface, inevitably with exposure to environmental hazards including particles, pathogens, and chemical irritants. Protective measures of the airways include anatomical barriers, ciliated epithelia, and mucus to trap and transport invaders (Ganesan et al., 2013), and a plethora of innate and adaptive immune cells throughout the airways. Barrier compromise leads to tissue injury, infection or multifactorial inflammatory disorders including asthma, COPD, and sarcoidosis. We first identified exosomes in healthy human bronchoalveolar lavage fluid (BALF), and found that they express the costimulatory molecule CD86, intercellular adhesion molecule 1 (ICAM-1, CD54) and MHC class II, and for most subjects also MHC Class I (Admyre et al., 2003). CD54 adheres to lymphocyte function associated antigen 1 (LFA-1) on antigen presenting cells (APC; Marlin and Springer, 1987), which together with CD86 suggested that pulmonary exosomes have immunomodulatory roles. Further, findings by us and others have linked lung inflammatory disorders to alterations in EV concentrations, cargo or function, and a picture is emerging of a role for EVs in inflammatory diseases.

## Potential cell sources of pulmonary EVs

The pulmonary EV cell sources are likely different during health and disease, but bronchial epithelial cells have been suggested as the main source of lung EVs (Kulshreshtha et al., 2013). This was based on the expression of CD63 or CD81 in epithelial cells, but for full certainty of the cellular origin the analyses should include detection of cell-specific markers in the exosomes. However, epithelial cells are at the front of environmental exposure, and bronchial epithelial cells have long been suggested to orchestrate pulmonary inflammation upon noxious stimuli (Cromwell et al., 1992), and it is likely that they respond to e.g., cigarette smoke with increased EV release. *In vitro*, interleukin (IL)-13 stimulation of epithelial cells released exosomes inducing proliferation of monocytic cells (Kulshreshtha et al., 2013), and decreasing EV release *in vivo* by GW4869, an inhibitor known to reduce EV release (Trajkovic et al., 2008), reduced disease burden in a murine asthma model (Kulshreshtha et al., 2013). Conversely, epithelial EVs may play protective roles as they carry mucins, glycoproteins vital for maintaining mucus barriers, and can bind to and neutralize influenza virus via mucin-contained alpha 2,6-sialic acids (Kesimer et al., 2009).

Macrophages are strongly increased in numbers in the lung in both COPD (Barnes, 2016) and Sarcoidosis (Zissel and Müller-Quernheim, 2015), and likely contribute to the pool of EVs. Alveolar macrophage-EVs transport suppressor of cytokine signaling (SOCS) proteins, and are taken up by alveolar epithelial cells, and inhibit IFNγ–induced activation of signal transducer and activator of transcription (STAT; Bourdonnay et al., 2015). This suggests a role for EVs in regulating pulmonary inflammatory processes (Bourdonnay et al., 2015). Exosomes from Mycobacterium-infected macrophages have inflammatory capacity (Bhatnagar and Schorey, 2007), and induce memory CD4^+^ and CD8^+^ T cell responses *in vivo* (Giri and Schorey, 2008). The authors reason that macrophage-released EV even represents a method for macrophages with low antigen presenting capacity to convey antigen-specific immune responses.

Endothelial MVs are affected during lung disease as a consequence of damage to lung capillaries. Circulating endothelial-derived MVs increase in smokers with signs of early emphysema (Gordon et al., 2011), and COPD patients have elevated endothelial MV numbers with increased expressions of endothelial markers (Takahashi et al., 2012). Other BAL fluid cells include eosinophils and neutrophils, which release cytokines and granules of strongly proinflammatory contents upon activation via pattern recognition receptors, Fc receptors, or cytokines (Barnes, 2008; Lambrecht and Hammad, 2015). Fernvik et al found that eosinophils have an intracellular pool enriched in the tetraspanin CD9, which was significantly reduced on activation (Fernvik et al., 1995), likely due to shedding of EVs which are enriched in CD9 (Kowal et al., 2014, 2016). Eosinophil-exosomes increase on stimulation with IFNγ, and asthma patient eosinophils release elevated exosome numbers with higher amounts of granule proteins (Mazzeo et al., 2015). Activated neutrophils release EVs carrying the granule protein myeloperoxidase (Hess et al., 1999), and can transport arachidonic acid to recipient platelets (Rossaint et al., 2016). Neutrophils communicate intensely with platelets via lipid mediators during inflammation, and mortality in *Escherichia coli*-induced murine lung inflammation was reduced by platelet uptake of neutrophilic EVs (Rossaint et al., 2016), speaking for an EV-based crosstalk between neutrophils and platelets in pulmonary inflammation.

Mast cell EVs can induce B and T cell proliferation (Skokos et al., 2001), dendritic cell (DC) maturation (Skokos et al., 2003), and production of anti-coagulant plasminogen activator inhibitor-1 (PAI-1) in endothelial cells (Al-Nedawi et al., 2005). Moreover, proteomic overlap of exosomes from lung patient tracheal aspirations and of exosomes from the human mastocytoma cell line-1 suggest that mast cell-EVs contribute to the pool of lung EVs (Veerappan et al., 2016).

Platelets regulate coagulation, and are also implicated in inflammation by interacting with leukocytes, and releasing proinflammatory granule contents (Herter et al., 2014). Platelet MVs aggravate LPS-induced damage of pulmonary endothelial cells (Xie et al., 2015), increase the adhesion of monocytes to endothelial cells (Barry et al., 1998), and induce IL-6 and IL-8 in an IL-1β dependent fashion in synovial joints of rheumatoid arthritis (RA) patients (Boilard et al., 2010).

APC-derived EVs can present antigen in a fashion that can induce antigen-specific immunogenicity (Raposo and Stoorvogel, 2013; Robbins and Morelli, 2014), and DC exosomes can display TLR ligands to other DCs leading to elevated tumor necrosis factor (TNF) secretion, increased DC-natural killer (NK) cell communication and elevated interferon (IFN)γ (Sobo-Vujanovic et al., 2014).

## EVs in lung diseases

**Asthma** can be allergic and induced by antigen-mediated hyperreactivity, or intrinsic and associated with repeated airway epithelial insults such as through cigarette smoke or infections, both leading to breathing difficulties due to bronchoconstriction, mucus overproduction, and airway remodeling (Lambrecht and Hammad, 2015). Asthma is orchestrated by IL-4-induced IgE-production and IL-5-induced expansion of eosinophils releasing proinflammatory and bronchoconstricting granula contents (Lambrecht and Hammad, 2015). We have shown that asthma BAL fluid exosomes display altered miRNA cargo including members of Let-7 miRNAs and the miR-200 family (Levänen et al., 2013), and we also found B-cell derived exosomes to carry allergenic peptides in an MHC context (Admyre et al., 2007). In contrast, BALF exosomes from allergen-exposed mice induced antigen-specific tolerance in recipient mice (Prado et al., 2008), demonstrating the flexible nature of EVs depending on the state of the producing cell. Leukotrienes (LT) are lipid mediators promoting mucus secretion, smooth muscle contraction and airway inflammation (Schauberger et al., 2016). Platelet MVs can transfer the LT-precursor arachidonic acid, and induce LT production in recipient platelets and endothelial cells (Barry et al., 1997). We found that human DC and macrophage exosomes with functional LT-forming enzymes induce granulocyte migration (Esser et al., 2010), and asthmatic patient exosomes induce inflammatory effects in bronchial epithelial cells including elevated LT and IL-8 production (Torregrosa Paredes et al., 2012). Importantly, this IL-8 production was blocked by a Cys-LT1 inhibitor.

**COPD** is initiated by long-term exposure to cigarette smoke or toxic irritants, followed by a response to tissue damage by DCs and macrophages releasing factors leading to airway remodeling, with a resulting reduction in lung capacity (Barnes, 2016). A small, but increasing, group of COPD patients are individuals associated with prenatal insults such as maternal cigarette smoke during pregnancy, or low birth weight caused by impaired development or pre-term birth (Stocks and Sonnappa, 2013). Cigarette smoke has profound effects on EV release. Mononuclear cells exposed to cigarette smoke extract (CSE) release MVs inducing production of IL-8, monocyte chemoattractant protein-1 and upregulation of CD54 in bronchial epithelial cells (Cordazzo et al., 2014). CSE-exposed epithelial cells release EVs enriched in the COPD-associated protein cysteine-rich angiogenic protein 61 (CCN1), and this increase contributes to the release of vascular endothelial growth factor and IL-8 (Moon et al., 2014). EVs from smoke-exposed bronchial epithelial cells induced differentiation of lung fibroblasts into myofibroblasts, suggesting that EVs may contribute to fibrotic development in COPD (Fujita et al., 2015). The glycoprotein alpha 1 anti-trypsin (A1AT) prevents excessive inflammation by inhibiting neutrophilic and eosinophilic enzymes, and A1AT deficiency leads to emphysema development (Stockley and Turner, 2014), a driver of COPD. EVs from pulmonary endothelial cells carry A1AT and may be involved in shuttling A1AT across the alveolar membrane to recipient epithelial cells (Lockett et al., 2014), possibly to prevent excessive pulmonary inflammation. In our recent investigations currently under review for publication, our preliminary findings showed alterations in the exosomal miRNA content due to both cigarette smoking and COPD, with significant gender difference due to disease. In COPD, dysregulation of exosomal miRNA was primarily observed in men. The observed down-regulations of a subset of miRNA occurred independently of current smoking, with significant correlations to the severity of disease in male, but not female COPD patients (the presentation from the ERS international congress 2016, London, is available online^1^).

**Sarcoidosis** is a multi-organ disease affecting the lungs in most patients, but also eyes, skin, spleen, nervous system, heart, kidney and liver may be affected. Pulmonary sarcoidosis is characterized by granuloma formation, pulmonary fibrotic development and functional impairment. Sarcoidosis has an unclear etiology, the most plausible explanation being a combination of genetic predisposition and non-resolved inflammation induced by pathogens or irritants leading to granuloma formation (Baughman et al., 2011). The mechanisms for multi-organ involvement are not clear, but dissemination of any pathogen involved, as well as aggravation of inflammation may be associated to EVs which exit pulmonary compartments reaching distant sites. We have identified exosomes in the BALF of sarcoidosis patients, and found a dramatic increase in exosome numbers, with more exosomal MHC Class I and II, tetraspanins as well as Heat shock protein(HSP)70 (Qazi et al., 2010). Compared to healthy subjects, patient exosomes induced more IL-13 and IFNγ production in autologous peripheral blood mononuclear cells (PBMC), and more IL-8 release by bronchial epithelial cells. Recently, our proteomic characterization of BALF exosomes from sarcoidosis patients revealed many proteins associated with inflammation and cellular migration, most strikingly an increase in most complement components (Martinez-Bravo et al., 2017). In addition, Vitamin D-binding protein (VDBP), a transporter of Vitamin D but also a potent chemoattractant for leukocytes, and precursor to a macrophage-activating factor, was significantly increased on both BALF and serum exosomes, which may be the result of pulmonary exosomes exiting into systemic circulation.

## Lung EVs in non-pulmonary disorders

Aside from their potential roles in pulmonary disease, we argue that lung EVs may be general disseminators of inflammation, with implications also for non-pulmonary disorders. In essence the airways represent an immunological frontline to pathogens and irritants, and airway cells exposed to e.g., cigarette smoke may respond by releasing EVs which in turn spread inflammation systemically. During inflammation, vascular permeability is dramatically increased (Nagy et al., 2008), with severe consequences for the integrity of the alveolar-capillary barrier in acute lung injury (Herold et al., 2013) and other pulmonary disorders. This will influence the exchange of EVs between the blood and the epithelial lining, with proinflammatory lung EVs reaching systemic circulation and potentially distant organs. In support of systemic spread, EVs have been shown to cross the blood-brain-barrier (BBB), and even seem to be the major transporter of a folate receptor from the choroid plexus (CP) into the cerebrospinal fluid (CSF; Grapp et al., 2013). On systemic LPS administration, the CP releases EVs inducing brain inflammation (Balusu et al., 2016), which is reduced by decreased EV release (Balusu et al., 2016). Exosomes have been used to deliver small interfering RNA across the BBB (Alvarez-Erviti et al., 2011), and to deliver curcumin with anti-inflammatory effects in the brain after nasal administration (Zhuang et al., 2011).

We hypothesize that airway cells thus convey pathogenic EVs to the circulation and thereby play yet undefined roles in other inflammatory diseases such as RA and multiple sclerosis (MS). In fact, smoking is the environmental factor most strongly linked to RA, and the early phase of RA has even been suggested to be initiated in the lungs after exposure to cigarette smoke or other irritants (Klareskog et al., 2009). Indeed, RA patients often present with immunological events preceding joint symptoms, possibly as a consequence of autoimmune events initiated in the lungs (as recently reviewed by Catrina et al. (2017). Furthermore, amongst smokers, the risk for MS is 50% higher and directly correlated to smoking frequency (Ascherio et al., 2012), thus MS is possibly also a disease with early events associated with pulmonary insult. EVs could be vehicles participating in this detrimental spread of inflammation.

Several studies have demonstrated the presence of pro-inflammatory EVs at the site of inflammation in RA (Berckmans et al., 2002; Song et al., 2005), MS (Marcos-Ramiro et al., 2014; Sáenz-Cuesta et al., 2014a,b), and inflammatory bowel disease (IBD; Mitsuhashi et al., 2016). MVs (Lindemann et al., 2001; MacKenzie et al., 2001) and exosomes (Qu et al., 2007) from several different cells are transporters of the strongly pro-inflammatory IL-1β, and membrane-bound TNF (mTNF) was found on synovial fibroblast exosomes from RA patients, but not from (non-autoimmune) osteoarthritis patients (Zhang et al., 2006). Synovial MVs from RA patients also promote coagulation, and bear markers of monocytic and granulocytic origin (Berckmans et al., 2002). In a murine autoimmune arthritis model, platelet-EV internalization by neutrophils was mediated by EVs carrying the lipid mediator enzyme 12-lipoxygenase (12-LO) combined with neutrophilic secreted phospholipase A2 IIA (sPLA2-IIA; Duchez et al., 2015). MS patients have increased serum levels of platelet MVs, which also correlate with clinical subtype (Sáenz-Cuesta et al., 2014a). MVs from MS patients disrupted endothelial barriers *in vitro* (Marcos-Ramiro et al., 2014), and EVs have been suggested to contribute to MS by degrading the blood brain barrier, increasing endothelial activation and promoting neural inflammation (Sáenz-Cuesta et al., 2014b). Fas ligand (FasL)-induced apoptosis is an immune regulatory mechanism, and DC-derived EVs with TNF, FasL and TNF related apoptosis inducing ligand (TRAIL) can activate natural killer cells, and induce tumor cell apoptosis (Munich et al., 2012), and could also play a role in systemic inflammation. In addition, the transcriptional regulator High mobility group box protein 1 (HMGB1), normally located in the cytosol, is released during necrosis and contributes to inflammation e.g., in acute lung injury (Kim et al., 2005), and one of the main exocytic routes of HMGB1 is via exosomes (Liu et al., 2006). HMGB1-exosomes from the lung could thus also contribute to inflammation systemically or locally at distant sites.

**Figure 1:**
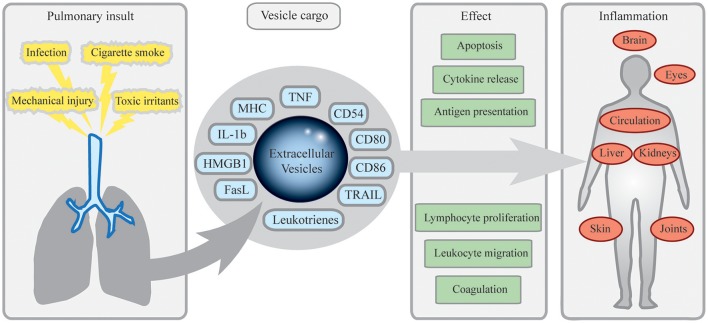
**Mechanisms of inflammatory dissemination by pulmonary extracellular vesicles (EV)**. Pulmonary insults including cigarette smoke, toxic irritants, infection, or mechanical injury such as burns or particle exposure lead to release of EVs from cells of the airways. Equipped with antigen presenting, costimulatory and pro-inflammatory molecules, these EVs may exit pulmonary compartments, spread systemically and induce cellular migration, apoptosis, cytokine release, and other effects contributing to inflammation. In peripheral lymphoid organs, this could induce antigen-specific reactions leading to inflammation and autoimmunity. Pulmonary EVs may also contribute to joint inflammation in rheumatoid arthritis, fever, and systemic effects via the central nervous system, spread of sarcoidosis to the eyes and the heart, and to dissemination of components from pathogens of the airways.

Airway EVs directly released from pathogens or APCs that have encountered pathogens, could also contribute to inflammation. *Pseudomonas aeruginosa* releases EVs with hemolysin and protease, suggested by the authors to play a role in bacterial sepsis (Kadurugamuwa and Beveridge, 1995). Also, *Mycobacterium avium* is an inhaled opportunistic pathogen infecting macrophages, which in turn release exosomes inducing TLR-dependent inflammation (Bhatnagar and Schorey, 2007). In a similar fashion, also patients with Inflammatory bowel disease (IBD) had intestinal proinflammatory EVs with elevated levels of IL-6, IL-8, and TNF on mRNA as well as protein level compared to healthy controls (Mitsuhashi et al., 2016). Connecting these systemic inflammatory EVs to the lung, pulmonary EVs can induce many of the effects discussed including adhesion, cellular migration and lipid mediator activities, as illustrated in Figure 1 and summarized in Table 1. Taken together, we propose that pulmonary EVs with pro-inflammatory cargo may contribute to spread of inflammation in both pulmonary and extrapulmonary disease.

**Table 1:** **Summary of EVs associated with inflammatory disease**.

**EV source**	**Cargo or effects**	**References**
Asthma patient BAL fluid exosomes	Display altered miRNA cargo including Let-7 and miR-200 members	Levänen et al., 2013
B cell exosomes	Carry MHC-associated allergenic peptides	Admyre et al., 2007
Platelet MVs	Transport arachidonic acid and induce leukotriene production	Barry et al., 1997
Human dendritic cell and macrophage exosomes	Induce granulocyte migration	Esser et al., 2010
Asthma patient exosomes	Trigger inflammation in bronchial epithelial cells	Torregrosa Paredes et al., 2012
Cigarette smoke exposed mononuclear cell MVs	Induce IL-8, chemotactic molecules, and CD54 increase in bronchial cells	Cordazzo et al., 2014
Cigarette smoke-exposed epithelial cell EVs	Enriched in COPD-associated protein and induce VEGF and IL-8	Moon et al., 2014
Smoke-exposed bronchial epithelial cell EVs	Induce myofibroblast differentiation	Fujita et al., 2015
Sarcoidosis patient BAL fluid exosomes	Higher MHC expression and elevated inflammatory effects	Qazi et al., 2010
Sarcoidosis patient BAL fluid exosomes	Many proteins associated with inflammation and migration	Martinez-Bravo et al., 2017
RA patient EVs	Proinflammatory EVs, membrane-TNF transporters	Berckmans et al., 2002; Song et al., 2005; Zhang et al., 2006
MVs and exosomes from multiple cell sources	General transporters of IL1-β	Lindemann et al., 2001; MacKenzie et al., 2001; Qu et al., 2007
Synovial MVs from RA patients	Promote coagulation	Berckmans et al., 2002
IBD patient EVs	Elevated levels of IL-6, IL-8 and TNF	Mitsuhashi et al., 2016

## Future challenges and concluding remarks

It is of importance to mention that EV research is still facing general challenges including finding the most suitable techniques for isolation and characterization of vesicles. Most commonly, EV isolation is based on differential centrifugations, which can be combined with density gradient separations to refine EV isolations. Alternative isolation methods include ultrafiltration and liquid chromatography, and it is important to carefully evaluate the choice of isolation method. Characterizing EVs involves a combination of methods, commonly including electron microscopy, flow cytometry and western blot detection of EV proteins (Théry et al., 2006). However, several markers previously considered exosome-associated including the tetraspanins CD9, CD63, and CD81 along with HSP70 (Raposo and Stoorvogel, 2013; Colombo et al., 2014) appear to be present in most EV categories (Kowal et al., 2016). Further, studying EV effects by specifically inhibiting their release or production is not possible as no universal EV-specific inhibitor has been reported so far. GW4869, an inhibitor of neutral sphingomyelases, reduces exosome production (Trajkovic et al., 2008), but is likely to also have other profound effects on the cells. Silencing of the small GTPase Rab27, implicated in exosome formation, reduces exosome-associated CD63, Alix, HSP70, and tumor susceptibility gene (TSG)101, but not CD9 or milk-fat globule-EGF factor 8 protein (MFGE8; Bobrie et al., 2012), pointing to a redundancy in exosome biogenesis.

Research based only on cell culture supernatants may oversimplify the *in vivo* functions of EVs due to the inherently limited milieu of cellular cooperation. Clinical samples provide the biological complexity necessary to study EVs, but are subject to experimental bias due to patient heterogeneity, and sample collection procedures. For BALF EV studies, large enough patient cohorts are necessary to reduce bias by individual variation, and importantly the BAL procedure should be performed prior to any biopsies to avoid blood contamination with plasma EVs in the BALF. Further, the volume and number of aliquots of instilled fluid, and the dwell time should preferably be constant between subjects, to minimize sampling differences. Another question is how representative BALF EVs are of the EVs present in the epithelial lining fluid and/or the tissues. Any differences in vascular permeability might lead to a difference in BALF EV profile, but this profile should still reflect the status of the lung, where more blood-derived EVs are likely to be found during inflammation.

EVs have the potential to initiate, aggravate and propagate inflammation, owing to their ability to ship proinflammatory molecules and to reach distant organs and compartments including the central nervous system. During inflammation there is a general increase in intercellular signaling with release of cytokines and lipid mediators regulating inflammatory activities, and EVs may be particularly potent. Each exosome and MV can transport a combination of inflammatory molecules, densely packed and even in combination with targeting molecules, antigen peptide-loaded MHC and costimulatory molecules. Furthermore, with the abilities to reach distant sites, EVs can expand communicative perimeters from inducing local to remote, or even systemic, effects. It is possible that EVs are central in mediating inflammation as they are major transporters of both TNF and IL1-β, so EV research has the potential of increasing understanding of fundamental inflammatory processes. With increasing evidence of EVs playing roles in pulmonary inflammatory disorders, we believe that they hold great potential in lung research to dissect pathogenesis, but also to identify biomarkers for disease. We further propose that inflammatory diseases affecting other specific organs or with systemic inflammation may be aggravated, or even initiated, by inflammatory EVs originating in and exiting from the lungs. Cigarette smoke, infection, injury and other insult to cells of the airways could lead to release of proinflammatory EVs. With the lungs being highly vascularized and on the border to the environment, they represent gates via which EVs can access systemic circulation and ship proinflammatory cargo. Further efforts are needed to study pulmonary EVs and their role in inflammation.

## Author contributions

All authors planned the manuscript, CW drafted it and all authors critically revised it.

## Funding

The authors are supported by the Swedish Medical Research Council (grant no. K2013-67x-15242-10-5), the Swedish Heart-Lung Foundation (grant no. 20140497), Hesselman's Foundation, the Stockholm County Council, the Cancer and Allergy Research Foundation, the Oscar II Jubilee Foundation, the Mats Kleberg Foundation, the Center for Allergy Research and the ChAMP consortium at the Karolinska Institutet, and the Karolinska Institutet.

### Conflict of interest statement

SG holds a patent for the use of exosomes in immune therapy. The other authors declare that the research was conducted in the absence of any commercial or financial relationships that could be construed as a potential conflict of interest.

## Footnotes

^1^ERS International Congress 2016, London, Session: Extracellular vesicles in lung disease pathogenesis, presentation ID 4771, http://www.ers-education.org/home/browse-all-content.aspx?idParent=156465
